# Hemophagocytic Lymphohistiocytosis Associated with *Salmonella typhi* Infection in a Child: A Case Report with Review of Literature

**DOI:** 10.1155/2018/6236270

**Published:** 2018-11-22

**Authors:** Juanita Uribe-Londono, Lina Maria Castano-Jaramillo, Laura Penagos-Tascon, Andrea Restrepo-Gouzy, Andres-Felipe Escobar-Gonzalez

**Affiliations:** ^1^Pediatric Department, Universidad CES, Medellin, Colombia; ^2^Pediatric Department, Universidad Pontificia Bolivariana, Medellin, Colombia; ^3^Pediatric Infectious Diseases, Pablo Tobon Uribe Hospital, Medellin, Colombia; ^4^Pediatic Hematology, Pablo Tobon Uribe Hospital, Medellin, Colombia

## Abstract

We present the case of an 8-year-old girl with hemophagocytic lymphohistiocytosis secondary to a *Salmonella typhi* infection. She received antibiotic treatment and intravenous immunoglobulin with complete resolution of the symptoms. We present a review of previously reported pediatric cases and propose a gradual approach to treatment.

## 1. Introduction

Hemophagocytic lymphohistiocytosis (HLH) is an infrequent but life-threatening syndrome due to excessive immune activation. It can occur as a primary disorder, caused by a genetic mutation, or as secondary sporadic cases triggered by infection, autoimmune diseases, or malignant diseases [1]. We report a case of secondary HLH in a girl with typhoid fever and review previously published pediatric cases.

## 2. Case

An 8-year-old girl, from a rural area in Choco (Colombian Pacific Coast), presented with 20 days of fever, hiporexia, asthenia, and arthralgias, associated with emesis and diarrhea. She was found to have anemia and was prescribed ferrous bisglycinate without improvement. The fever was persistent and was associated with chills, abdominal pain, and dark urine; she was admitted to a rural hospital and diagnosed with pancytopenia; 2 units of packed red blood cells were transfused; and she was transferred to our hospital with a clinical suspicion of lymphoproliferative disorder. Her vital signs showed tachycardia (131 bpm), tachypnea (45 per min), and limit-low oxygen saturation (91%). Physical examination revealed jaundice and hepatosplenomegaly of 6 cm and of 2 cm below the costal margin, respectively; fine right basal crackles and a soft systolic heart murmur were heard at the left sternal border and the third intercostal space. Her past medical history was relevant for posttraumatic osteomyelitis of the right humerus and septic arthritis of the right elbow; her parents were not consanguineous, and she did not have pseudoalbinism.

Test results revealed thrombocytopenia and lymphopenia (Hg 11.5 g/dL, WBCs 4600/*µ*L, neutrophils 3404/*µ*L, lymphocytes 1058/*µ*L, and platelets 59000/*µ*L), elevated C-reactive protein (21.57 mg/dL), and altered liver function tests (ALT 349 U/L, AST 135 U/L, total bilirubin 6.67 mg/dL, direct bilirubin 5.3 mg/dL, LDH 1376 IU/L, and albumin 2.1 g/dL). Infectious workup was negative for HIV, dengue, malaria, Hepatitis B, Hepatitis C, CMV, EBV, and *Mycoplasma*. Additional laboratory tests showed hypertriglyceridemia (787 mg/dL) and hyperferritinemia (>2000 ng/mL) with normal fibrinogen (311 mg/dL). Echocardiogram showed minimal pericardial effusion and preserved ventricular function without anatomic defects or signs of endocarditis. Abdominal ultrasound showed a small right pleural effusion, hepatomegaly, splenomegaly, and retroperitoneal and hepatic hilar lymphadenopathies. Abdominal computed tomography showed the same findings, with hypodensity areas corresponding to splenic infarcts. She was diagnosed with hemophagocytic lymphohistiocytosis and started on piperacillin-tazobactam and intravenous immunoglobulin (1 g/kg/d for 2 days); while waiting for blood cultures and bone marrow analysis, CSF was within normal limits. On her third hospital night, she presented hematemesis and rectorrhagia with hypovolemic shock that required fluid resuscitation, intravenous infusion of omeprazole, tranexamic acid, phytomenadione, packed red blood cell, and platelets transfusion. She was stabilized; upper and lower endoscopies did not show signs of active bleeding, but just some residual melena. On the fourth day of hospitalization, both blood and bone marrow cultures were positive for *Salmonella typhi*; and the antibiotic regimen was changed to ciprofloxacin; bone marrow specimen showed hematopoietic precursors with no signs of malignancy; and no hemophagocytosis was appreciated. Repeated blood cultures were negative; during the hospital stay, she was started on enalapril to maintain a tight control on blood pressure. She completed 14 days of antibiotic treatment with resolution of the fever, hepatomegaly, splenomegaly, and the abdominal pain. Laboratory investigations before discharge showed resolution of the cytopenias, down trending of liver function tests and triglycerides levels, and ferritin was still elevated.

## 3. Discussion

Infections are potential triggers for primary and sporadic HLH cases. Viruses are the most common cause, but bacterial, fungal, parasitic, and tropical infections have also been associated [1]. Secondary HLH as a complication of typhoid fever by *Salmonella typhi* has been previously described in adults; to the best of our knowledge, this is the sixth case described in the pediatric population (Table 1) [2–6]. In children, the most prominent characteristics are fever, splenomegaly, neurologic manifestations, anemia, and thrombocytopenia, those frequently require transfusion support. Abdominal pain and hepatitis are common, with a higher elevation of aspartate transaminase. Clinical response to antibiotics is excellent, with all the cases improving after 5 or 6 days of antimicrobial therapy, without the need of HLH-specific medications such as immunosuppressants or chemotherapeutic agents.

The absence of hemophagocytosis does not rule out the diagnosis, and in our case, other diagnostic criteria were met; hemophagocytosis was not evident in the initial bone marrow analysis, and since malignancy was ruled out and the patient improved, no repeated bone marrow studies were done.

None of the four pediatric cases reported in the literature had NK-cell activity and soluble IL-2 (CD25) receptor tests; in Colombia, they are not widely available, so the diagnosis of HLH in the setting of a typhoid fever is challenging. Fever and splenomegaly are hallmarks of *Salmonella typhi* infection, and hepatitis and cytopenias may be seen secondary to bone marrow suppression [6]. In the setting of infection-associated HLH, a higher cutoff value for ferritin is reasonable, 2000 ng/ml rather than the 500 ng/ml suggested by the HLH-2004 criteria, since the infection per se can cause hyperferritinemia [7, 8].

Some of the case reports do not fulfill 5 of the 8 actual criteria for HLH [7]. Alternative modified criteria have been proposed, with at least 3 out of 4 clinical manifestations (fever, splenomegaly, cytopenias of minimum 2 lines, and hepatitis) and at least 1 out of 4 laboratory criteria (hemophagocytosis, hyperferritinemia, increased soluble IL-2 (CD25) receptor, and absent or very decreased NK function), with hypertriglyceridemia, hypofibrinogenemia, and hyponatremia supporting the diagnosis [9]. When these modified criteria are considered, all the reported cases are classified as HLH. In our case, no hemophagocytosis was evident in the bone marrow, but other laboratory criteria were met.

The initial conduct for infection-associated HLH is treating the triggering cause, since in most patients this will be enough to withdraw the immune activation stimulus and control the inflammatory cytokine storm [9], as seen in most of the pediatric cases of HLH secondary to *Salmonella typhi* (Table 1). However, in acutely ill or deteriorating patients, immunomodulatory therapy for HLH may be needed. In our case, the patient had signs of systemic inflammatory response and generally unwell appearance, so IVIG (intravenous immunoglobulin) was used early in the course of the disease.

Specific immunomodulatory therapy for HLH can be based on the recommendations by the Histiocyte Society (HLH-2004); this protocol uses high-dose antineoplastic and immunosuppressant schemes. IGIV can be used in secondary HLH, as it has been shown to be as effective as the HLH-2004 protocol, with fewer adverse effects [10].

**Figure 1:**
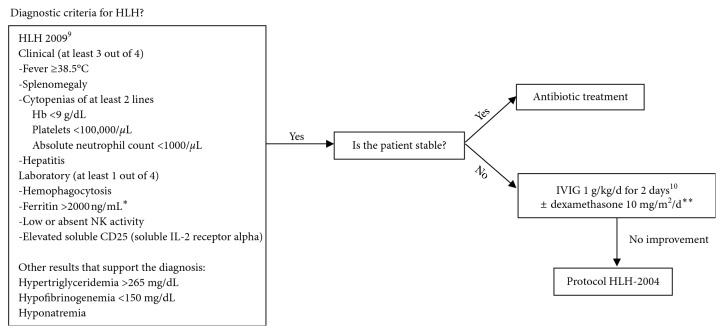
Approach to HLH secondary to *Salmonella* infection. IVIG = intravenous immunoglobulin; ^*∗*^value suggested by authors; in the original diagnostic criteria, ferritin is >500 ng/mL; ^*∗∗*^dexamethasone 10 mg/m/d for 7 days followed by 6 mg/m^2^/d until complete response.

In our experience, treating the trigger as a first-line measure might be an effective measure. Patients with a torpid evolution or who are acutelly ill may benefit from IVIG +/− steroids to control the immune response; leaving the HLH-2004 protocol as a last resort for patients who do not improve despite the previous treatments (Figure 1).

## Figures and Tables

**Table 1:** Characteristics or reported pediatric cases of HLH secondary to typhoid fever.

	This case report (Colombia)	Fame et al. [4] (USA)	Chien et al. [5] (Taiwan)	Caksen et al. [6] (Turkey)	Runel-Belliard et al. [2] (Comoros)	Pandey et al. [3] (India)
Patient	8-year-old girl	13-year-old girl	13-year-old boy	6-year-old boy	7-year-old boy	10-year-old boy
Fever	20 days	14 days	7 days	10 days	8 days	5 days
Hepatomegaly/splenomegaly	+/+	+/+	+/−	−/+	−/+	+/+
Other clinical findings	Abdominal pain, jaundice, right pleural effusion, upper GI bleed	Somnolence	Psychosis, brain edema, maculopapular rash	Abdominal pain, jaundice, headache, hyponatremia	Dysenteric diarrhea, abdominal pain, hyponatremia	Meningeal signs
Hemoglobin (g/dL)	5.8	7.7	10.6	6.9	7.3	6.9
WBC (cells/*μ*L)	2.590	3.300	2.940	3.400	1.300	800
Neutrophils (cells/*μ*L)	1.445	2.145^a^	2.499	—	700	—
Platelets per *μ*L	101.000	20.000	20.000	48.000	18.000	10.000
Transfusion support required	Yes	No	No	No	Yes	Yes
AST/ALT (U/L)	349/135	160/31	746/–	433/98	—	—
Ferritin (ng/mL)	>2.000	—	—	—	—	1791
Fibrinogen (mg/dL)	311	—	—	—	105	—
Triglycerides (mg/dL)	787	—	350	—	251	265
Hemophagocytosis	No	Yes	Yes	Yes	—	Yes
Soluble IL-2 receptor	—	—	—	—	—	—
NK activity	—	—	—	—	—	—
Treatment	Ciprofloxacin IVIG	Ampicillin TMP-SMZ^b^	Ceftriaxone	Chloramphenicol	Ceftriaxone fluoroquinolone	Ceftriaxone
Time to improvement	5 days	5 days	3 days^c^	—	6 days	5 days

WBC = white blood cells; AST = aspartate transaminase; ALT = alanine transaminase; IL-2 = interleukin 2; NK = natural killer; IVIG = intravenous immunoglobulin; TMP-SMZ = trimethoprim sulfamethoxazole. ^a^First course of ampicillin for 10 days, relapse with the same strain. ^b^First course with ampicillin for 10 days, relapse with the same strain treated with TMP-SMZ for 14 days. ^c^Neurologic symptoms improved after 3 days, fever after 10 days.
